# Prevalence and predictors of nomophobia among the general population in two middle eastern countries

**DOI:** 10.1186/s12888-022-04168-8

**Published:** 2022-08-02

**Authors:** Hassan Alwafi, Abdallah Y. Naser, Abdulelah M. Aldhahir, Alaa Idrees Fatani, Rahaf Awaili Alharbi, Khawlah Ghazi Alharbi, Braah Ali Almutwakkil, Emad Salawati, Rakan Ekram, Mohammed Samannodi, Mohammed A. Almatrafi, Wael Rammal, Hamza Assaggaf, Jumanah T. Qedair, Abdullah A. Al Qurashi, Afnan Alqurashi

**Affiliations:** 1 Department of Medicine, Faculty of Medicine, Umm Al-Qura University, Makkah, Saudi Arabia; 2 Department of Applied Pharmaceutical Sciences and Clinical Pharmacy, Faculty of Pharmacy, Isra University, Amman, Jordan; 3 Respiratory Therapy Department, Faculty of Applied Medical Sciences, Jazan University, Jazan, Saudi Arabia; 4 Department of Medicine and Surgery, Faculty of Medicine, Umm Al-Qura University, Makkah, Saudi Arabia; 5 Department of Family Medicine, Faculty of Medicine, King Abdulaziz University, Jeddah, Saudi Arabia; 6 School of Public Health and Health Informatics, Umm Al-Qura University, Mecca, Saudi Arabia; 7 Department of Pediatrics, Umm Al-Qura University, Makkah, Saudi Arabia; 8 Department of Surgery, King Abdulaziz Medical City, Jeddah, Saudi Arabia; 9 Department of Laboratory Medicine, Faculty of Applied Medical Sciences, Umm Al-Qura University, Makkah, Saudi Arabia; 10 College of Medicine, King Saud Bin Abdulaziz University for Health Sciences, Jeddah, Saudi Arabia; 11 King Abdullah International Medical Research Center, Jeddah, Saudi Arabia; 12 Independent researcher, Mecca, Saudi Arabia

**Keywords:** Anxiety, Jordan, Mental disorder, Middle East, Nomophobia, Saudi Arabia

## Abstract

**Background:**

Nomophobia is a psychological condition caused by a fear of disconnecting from others through mobile phones.

**Aim:**

This study aims to determine the prevalence of and predictors of nomophobia and anxiety symptoms among the general population in Saudi Arabia and Jordan.

**Methods:**

This study was an observational cross-sectional study using a web-based online survey distributed in two middle eastern countries (Saudi Arabia and Jordan) between Jun 24 and Jul 20, 2021. A convenience sample was used to recruit the study participants. Categorical variables were identified as frequencies and percentages. In addition, a binary logistic regression analysis was used to determine the factors associated with nomophobia symptoms. The Statistical Package for Social Science (SPSS) software, version 27 (IBM Corp, Armonk, NY, USA), analyzed the data.

**Results:**

A total of 5,191 responded to the online survey. Around (26.5%) reported that they suffer from an anxiety problem or use a treatment for anxiety. The median daily time spent using a mobile phone (IQR) (minutes) was around 210 min per day. About half of the study sample (51.2%) are diagnosed with dependence syndrome. The binary logistic regression analysis revealed that those within the age group of 30–49 years and 50 years and above) are less likely to have mobile phone dependence compared to those less than 30 years old. Females were 16% at lower risk of developing mobile phone dependence compared to males Married participants were less likely to have mobile phone dependence compared to single participants (OR: 0.62 (95% CI 0.56–0.70)), while divorced participants were at a 46% higher risk of developing mobile phone dependence.

**Conclusion:**

Nomophobia prevalence among Saudi Arabia and Jordon's population is 51.2%. Several factors may predict mobile phone dependence including age, gender, marital status, and previous history of anxiety.

## Background

Anxiety disorders are among the most prevalent mental illnesses worldwide and in the middle east [1]. It is associated with other mental conditions, such as depression, substance abuse, and a higher suicidal rate [2]. It is also associated with elevated cardiovascular risk factors and a higher rate of cardiovascular diseases [3]. Anxiety is a significant burden for many societies. Globally, the estimated cost of scaling up treatment, primarily psychosocial counseling and antidepressant medication, amounted to US$ 147 billion [4]. According to a systematic review and a meta-analysis conducted in 2013, the prevalence of anxiety disorders ranged from 0.9% to 28.3% [5]. In Saudi Arabia, anxiety disorders account for 12.3%, which is the most common disorder [6].

Smartphones have become a standard tool to help people in their regular daily activities [7]. More than 200 virtual social networks have developed and exist today, including Facebook, Twitter, and Instagram [8]. Due to excessive smartphone utilization, people are susceptible to unlimited psychological problems, such as Nomophobia [9]. Nomophobia is a psychological condition caused by a fear of disconnecting from others through mobile phones [10]. It can also be defined as the fear of not being able to utilize the services and applications of the mobile phone, and not being able to communicate to others [11]. Nomophobia is also associated with a lower self-esteem and extrovert personality [10]. Nomophobia may also exacerbate other mental disorders, such as a social phobia or social anxiety [10]. Factors such as increased in the duration of using smartphones, daily usage time, daily frequency of checking smartphones, and daily mobile internet usage time may increase the level of anxiety and fear of disconnecting from mobile phones.

Globally, The prevalence of Nomophobia was 70.76% for moderate to severe cases and 20.81% for severe cases, with university students being the most affected group [12]. In the United Kingdom (UK), the UK post office reported that around 53% of the population suffer from anxiety and fear when disconnected from their smartphone [13]. Similarly, other studies reported a high prevalence rate of nomophobia among students and health care professionals [14–16]. However, data on the prevalence and factors associated with Nomophobia in the middle east among the general population are lacking. Accordingly, this research aimed to determine the prevalence of and predictors of nomophobia and anxiety symptoms among the general population in Saudi Arabia and Jordan.

## Methods

### Study design and study population

This study was an observational cross-sectional study using a web-based online survey distributed in two middle eastern countries (Saudi Arabia and Jordan) between Jun 24 and Jul 20, 2021.

### Sampling strategy

A convenience sample of eligible participants was used to recruit the study participants. Participants were invited to participate in this study through social media (Facebook, Twitter, Snapchat, and Instagram). The study sample was invited using a survey link. Since all participants volunteered for the study, they were not required to provide written informed consent. Study aims and objectives were clearly explained at the beginning of the invitation letter of the survey.

The inclusion criteria were participants aged 18 years and above and living in Saudi Arabia or Jordan.

The survey link was re-posted weekly to increase response and make the survey more accessible to the general population.

### Questionnaire tool

A previously developed questionnaire by Aggarwal et al. was used to achieve the study objectives [17]. The original survey included 20 questions, and its purpose was to gather data on mobile usage patterns and determine whether or not they matched the ICD-10 criteria for substance dependence syndrome. The first three questions asked about the number of years spent using a mobile phone, the average daily usage time, and the reason for use. The remaining 20 questions were in the form of a questionnaire with a binary (yes/no) response option that inquired about respondents' mobile usage habits and whether they fulfilled the ICD-10 criteria for dependent syndrome. Six ICD-10 dependency syndrome criteria were addressed by 14 of the 20 items (one question for intense desire, four questions for impaired control, three questions for withdrawal, one question for tolerance, four questions for decreased pleasure, and one question for harmful use). If a participant responded "yes" to all of the questions in a single-question criteria or "yes" to at least half of the questions in multiple-question criteria, they were considered to have met the criterion. If three or more of the ICD-10 dependent criteria were met by the participants, they were considered to have a mobile phone dependence. The 20 items that addressed mobile dependence were scored by giving one point to each positive (yes) response from participants to determine how dependent they were on their mobile devices. The higher the score the more dependent on their mobile device. The questionnaire was translated into Arabic using the forward–backward method and then used in this study.

### Sample size

The target sample size was estimated based on the WHO recommendations for the minimal sample size needed for a prevalence study [18]. This study used a confidence interval of 95%, a standard deviation of 0.5, and a margin of error of 5%. The required sample size was 385 participants.

### Ethical statement

The Research Ethics Committee approved this study at Umm Al Qura University, College of Medicine. Participants were informed that completing the questionnaire is considered as written consent to participate.

### Statistical analysis

The (SPSS) Statistical Package for Social Science software, version 27 (IBM Corp, Armonk, NY, USA), was used to analyze the data. Categorical variables were identified as frequencies and percentages. A binary logistic regression analysis was used to determine the factors associated with nomophobia symptoms. The cut-off point for the logistic regression was fulfilling three or more of the ICD-10 criteria for substance dependence syndrome. A confidence interval of 95% (P 0.05) was used to determine the statistical significance of the results, with a predetermined level of significance of 5%

## Results

### Demographics characteristics

A total of 5,191 responded to the online survey. Most of the participants were from Saudi Arabia (85%). More than half of the study participants (69%) were females aged below 50 years (87%). Around 66.5% reported holding a bachelor's degree. About half of the study participants (52.9%) were single, while 42.3% were married; however, more single participants (57.4%) were included in the Saudi Arabia sample than in the Jordanian sample (26.7%). Around (26.5%) reported that they suffer from an anxiety problem or use a treatment for anxiety. The median daily time spent using a mobile phone (IQR) (minutes) was around 210 min per day. Table 1 below illustrates the demographic background of the study participants.

**Table 1:** Participants demographic characteristics

Demographic variable	Overall (*n* = 5191)		Saudi Arabia (*n* = 4422)		Jordan (*n* = 769)	
	f (%)		Frequency	Percentage	Frequency	Percentage
Age categories (years)						
18–29 years	2998 (57.8%)		2781	62.9%	217	28.2%
30–49 years	1515	29.2%	1200	27.1%	315	41.0%
50 years and above	678	13.1%	441	10.0%	237	30.8%
Gender						
Females	3582	69.0%	3025	68.4%	557	72.4%
Marital status						
Single	2745	52.9%	2538	57.4%	207	26.9%
Married	2196	42.3%	1683	38.1%	513	66.7%
Divorced	163	3.1%	145	3.3%	18	2.3%
Widowed	87	1.7%	56	1.3%	31	2.3%
Education level						
Secondary school level	1410	27.2%	1224	31.9%	186	26.2%
Bachelor degree	3453	66.5%	2971	67.2%	482	62.7%
Higher education	328	6.3%	227	5.9%	101	14.2%
Employment status						
Retired	311	6.0%	215	5.6%	96	13.5%
Unemployed	1161	22.4%	881	22.9%	280	39.5%
Employed	1956	37.7%	1643	37.2%	313	40.7%
Students	1763	34.0%	1683	43.8%	80	11.3%
Income level						
Less than 700$	2658	51.2%	2254	51.0%	404	52.5%
700–1500$	741	14.3%	507	11.5%	234	30.4%
1500–2100$	388	7.5%	322	7.3%	66	8.6%
2100$ and above	1404	27.0%	1339	30.3%	65	8.5%
In general, do you suffer from an anxiety problem or use a treatment for anxiety?						
Yes	1378	26.5%	1184	27.2%	194	28.4%
Median daily time spent on using mobile phone (IQR) (minutes):						
	210.0 (IQR: 180.0)		210 (IQR: 150.0)		140.0 (IQR: 108.5)	
Duration of mobile phone usage (years):						
Less than one year	45	0.9%	30	0.7%	15	2.0%
1–5 years	506	9.7%	429	9.7%	77	10.0%
6–10 years	1700	32.7%	1489	33.7%	211	27.4%
10 years and above	2940	56.6%	2474	55.9%	466	60.6%

### Mobile phone use pattern

Most of the study participants reported that they lose track of time after starting to use mobile phones for (SMS, games, or music) (71.9%), and (73.3%) get irritated in the morning if they do not locate their mobile phone. Around half of the sample size (49.3%) reported that mobile phone use has made them spend less time with friends or family. In addition, around (42.2%) reported that they become anxious about missing something when they have to switch off their mobile phone for some reason. However, around (56%) reported that using a mobile phone helps them overcome bad moods (e.g. feeling of inferiority, helplessness, guilt, anxiety, and depression). Details of the pattern of the study participants' responses regarding their mobile use pattern are listed in Table 2.

**Table 2:** Participants response to the questionnaire items

Question	Yes
Q1. When not using the mobile, are you preoccupied with the mobile phone (Keep constantly thinking about the previous and the future uses)?	41.9%
Q2. Do you need to use mobile phone for increased amounts of time in order to achieve satisfaction/betterment?	40.7%
Q3. Have you made unsuccessful efforts to control/decrease or stop mobile phone use?	46.0%
Q4. Do you get upset when attempting to cut down mobile phone use?	32.0%
Q5. Has mobile phone use led to decrease in meeting the friends in person?	39.1%
Q6. Has mobile phone use has made you spend less time with friends/ family?	49.3%
Q7. Has mobile phone use has led to decrease in socialization? (meeting friends/ hanging out)	35.4%
Q8. Do you lose track of time after starting to use mobile phone for SMS, games, music etc.?	71.9%
Q9. Do you lie to others to conceal the extent of your use of mobile phone?	19.1%
Q10. Do you become anxious of missing something if you have to switch off your mobile phone for some reason?	42.4%
Q11. Do you compulsively respond to calls/ SMSs at places which don’t permit (Class, driving, group participation)?	32.6%
Q12. Do you compulsively respond to calls/ SMSs at places where it is dangerous to do so (crossing road, driving/ working at machines)?	23.7%
Q13. Do you call back to most of the missed calls?	54.5%
Q14. Does using mobile phone help you to overcome the bad moods (e.g. feeling of inferiority, helplessness, guilt, anxiety, depression etc.)?	56.8%
Q15. Do you feel guilty about the expenditure on (or excessive use of) mobile phone?	42.1%
Q16. Do you get irritated in the morning if you are not able to locate your mobile phone?	73.3%
Q17. Do your families/ friends/ colleagues complain that your mobile phone use is excessive?	34.0%
Q18. Do you get annoyed or shout if someone asks you to decrease the use of mobile phone?	22.7%
Q19. Do you frequently participate in SMSs or phone entry competitions?	17.2%
Q20. Do you think you are getting addicted to mobile use?	49.8%

### Participants' mobile dependence score

The mean mobile phone dependence score for the Jordanian participants was lower than that for the Saudi population with 7.05 (± 4.00) compared to 8.49 (± 4.63). Details of the participants' mean mobile phone dependence scores stratified by their demographic characteristics are listed in Table 3.

**Table 3:** Participants mean mobile phone dependence score stratified by their demographic characteristics

Demographic variable	Overall		Saudi Arabia		Jordan	
	Mean ( ±)		Mean	SD	Mean	SD
Age categories (years)						
18–29 years	8.93 ± 4.54		8.97	4.57	8.40	4.06
30–49 years	7.84	4.55	8.08	4.67	6.95	3.96
50 years and above	6.21	4.03	6.38	4.23	5.89	3.60
Gender						
Males	8.53	4.74	8.68	4.78	7.67	4.43
Females	8.18	4.50	8.42	4.57	6.81	3.80
Marital status						
Single	8.95	4.53	9.00	4.56	8.29	4.13
Married	7.45	4.42	7.68	4.55	6.65	3.88
Divorced	8.85	5.31	9.05	5.40	7.38	4.54
Widowed	6.75	4.46	7.46	4.99	5.52	3.05
Education level						
Secondary school level	8.66	4.61	8.92	4.63	6.92	4.04
Bachelor degree	8.13	4.53	8.30	4.60	7.04	3.94
Higher education	7.93	4.68	8.15	4.84	7.36	4.20
Employment status						
Retired	6.14	4.11	6.35	4.23	5.61	3.79
Unemployed	7.93	4.59	8.31	4.77	6.68	3.68
Employed	8.37	4.77	8.53	4.88	7.61	4.16
Students	8.81	4.36	8.84	4.37	8.34	4.18
Income level						
Less than 700$	8.46	4.44	8.72	4.45	6.91	4.05
700–1500$	8.09	4.61	8.46	4.84	7.28	3.94
1500–2100$	8.46	4.55	8.64	4.69	7.44	3.53
2100$ and above	7.93	4.83	7.99	4.85	6.80	4.30
In general, do you suffer from an anxiety problem or use a treatment for anxiety?						
No	7.73	4.46	7.99	4.52	6.17	3.65
Yes	9.75	4.56	9.84	4.65	9.26	3.98
Duration of mobile phone usage (years):						
Less than one year	9.04	5.20	9.36	5.73	8.38	3.93
1–5 years	8.68	4.58	8.91	4.59	7.39	4.30
6–10 years	8.57	4.39	8.71	4.41	7.55	4.06
10 years and above	8.02	4.66	8.26	4.75	6.71	3.89

### ICD-10 criteria for mobile dependence syndrome

Table 4 presents the study participants who fulfil the ICD-10 criteria for mobile dependence syndrome. About half of the study sample (51.2%) are diagnosed (symptomatically according to ICD-10 criteria for dependence syndrome). The most common dependence criteria across the study sample were withdrawal and impaired control (60.1%) and (55.5%), respectively. The harmful use was the least common (23.7%).

**Table 4:** ICD-10 diagnostic criteria for mobile dependence syndrome

ICD-10 Criteria for Dependence syndrome	Percentage of participants
Intense Desire (Q1)	41.9%
Impaired control (Q3,8,11,19)	55.5%
Withdrawal (Q10,13,16)	60.1%
Tolerance (Q2)	40.7%
Decreased pleasure (Q5,6,7,17)	45.9%
Harmful use (Q12)	23.7%
Dependence syndrome among Mobile phone users	51.2%

### Risk factors of mobile phone dependence

The binary logistic regression analysis revealed that those within the age group of 30–49 years and 50 years and above) are less likely to have mobile phone dependence compared to those less than 30 years old (OR: 0.85 (95% CI 0.76–0.96), *p* < 0.01), and (OR: 0.44 (95% CI 0.37–0.52), *p* < 0.001). Females were 16% at lower risk of developing mobile phone dependence compared to males (Odds ratio (OR): 0.84 (95% CI 0.75–0.95), *p* ≤ 0.01). Married participants were less likely to have mobile phone dependence compared to single participants (OR: 0.62 (95% CI 0.56–0.70), *p* < 0.001), while divorced participants were at a 46% higher risk of developing mobile phone dependence (OR: 1.46 (95% CI 1.06–2.00), *p* < 0.05). Participants who have reported having anxiety problems or using a treatment for anxiety were at higher risk of developing mobile phone dependence by 75% (OR: 2.17 (95% CI 1.91–2.47), *p* < 0.001), Table 5.

**Table 5:** Binary logistic regression analysis

Demographic variable	Odds ratio of being mobile phone dependent (95%CI)
Age categories (years)	
18–29 years (Reference group)	1.00
30–49 years	0.85 (0.76–0.96) ^**^
50 years and above	0.44 (0.37–0.52) ^***^
Gender	
Males (Reference group)	1.00
Females	0.84 (0.75–0.95) ^**^
Marital status	
Single (Reference group)	1.00
Married	0.62 (0.56–0.70) ^***^
Divorced	1.46 (1.06–2.00)*
Widowed	0.52 (0.34–0.81) ^**^
Education level	
Secondary school level (Reference group)	1.00
Bachelor degree	0.87 (0.78–0.97) ^*^
Higher education	0.89 (0.71–1.12)
Employment status	
Retired (Reference group)	1.00
Unemployed	0.82 (0.72–0.94) ^**^
Employed	0.96 (0.85–1.09)
Students	1.37 (1.22–1.54) ^***^
Income level	
Less than 700$ (Reference group)	1.00
700–1500$	0.93 (0.79–1.08)
1500–2100$	0.98 (0.80–1.21)
2100$ and above	0.90 (0.79–1.01)
In general, do you suffer from an anxiety problem or use a treatment for anxiety?	
No (Reference group)	1.00
Yes	2.17 (1.91–2.47) ^***^
Duration of mobile phone usage (years):	
Less than one year (Reference group)	1.00
1–5 years	1.20 (1.00–1.45) ^*^
6–10 years	1.20 (1.07–1.34) ^**^
10 years and above	0.79 (0.71–0.89) ^***^

^*^ *p* < 0.05

^**^ *p* < 0.01

^***^ *p* < 0.001

## Discussion

The COVID-19 epidemic has impacted the mental health of a wide range of people, including the general public, healthcare professionals, and university students. All studies agree that the epidemic has increased people's stress levels, increasing their risk of developing anxiety, depression, and other mental illnesses [19–27]. This study was a cross-sectional web-based survey in Saudi Arabia and Jordan. It aimed to determine the prevalence and factors associated with nomophobia in the general population. The result showed similar proportions of people who suffer from anxiety problems or use a treatment for anxiety in Saudi Arabia (26.5%) and Jordan (28.4%).

ICD-10 criteria for mobile phone syndrome demonstrated that around half of the sample (51.2%) are diagnosed with mobile phone dependence. According to the requirements (60.1%) were withdrawn, and (55.5%) had impaired control. Additionally, our study discovered that 73.3% of the participants developed a fear of being without their mobile phones and that approximately 71.9% spent time on their phones for SMS, games, or music, which could be attributed to the exceptional circumstance of the coronavirus (COVID-19) pandemic in 2019. All governments tried to identify and use new digital communication because all venues and local businesses were shut down, and national lockdowns were imposed [28].

The median daily time for phone use in Saudi Arabia was (210 min), while in Jordan was (140 min). So, compared to Jordan, people in KSA more frequently use mobile phones, maybe due to the COVID19 pandemic and the 2030 vision that aimed to use electronic communication and information technology [29]. Moreover, the Saudi population's mean mobile phone dependence score was higher than that of the Jordan population with 8.49 (SD: 4.63) compared to 7.05 (SD: 4.00). Thus, it may increase anxiety to be continually connected to their mobile phone [30].

Our results showed that the students also are more likely to have mobile phone dependence. It could be due to the impact of COVID-19 to continue tutoring and assessing via virtual classes [31]. According to our study's findings, the younger age (18–29) year appear to be at higher risk of developing mobile phone dependence than other age groups; these findings are consistent with the previous study conducted in 2019 [32], and these results might be explained by the fact that young people are more familiar with modern technologies and tools than people of other ages [33]. For example, whereas people above 30 are less likely to get mobile phone dependence, it could be that they are preoccupied with other things such as work and raising children [33].

Duration of mobile phone use compared to India in between 18–29 years of age group, in KSA the majority were using mobile phone for more than ten years 55.9%, and 60.6% in Jordan, on the other hand only 13.29% in India [34]. This points out that the use of phones in Saudi Arabia and Jordan was earlier in age compared to India. Another study conducted in Bangalore reported that the mobile phone addiction level in people who used the mobile phone under thirteen years was higher than in the older age group [35]. Therefore, the increased number of years of use of mobile phones is highly associated with mobile dependence. So, it could be one of the risk factors for developing nomophobia among this age group.

Another exciting finding in our sample is that multivariate effects results indicate that gender effect on nomophobia was statistically significant, and the male gender tends to have a higher risk of mobile dependence than females. This is similar to the findings reported by the previous studies [32, 36]. However, this is inconsistent with other studies in the literature where women are more likely to suffer from nomophobia in many countries like Turkey and India [32, 37]. Also, a survey in Bahrain detects more severe Nomophobia in females (21.7%) rather than males (18.8%) [38]. In contrast, some previous studies showed no significant gender differences [31, 34]. Gender differences may be due to men believing that mobile phone technology increases their independence. At the same time, women use mobile phones primarily for communication, social networking, and staying connected with friends and family, which could explain why females are less likely to have mobile dependence [32].

A study in Brazil [39] showed that 68% of total participants reported mobile phone dependency. Participants with panic disorder would have significantly more emotional symptoms and reliance on mobile phones than the control group if access to their mobile phones were restricted. In our study, the binary logistic regression results also showed that participants who have reported having anxiety problems or using a treatment for anxiety were at a higher risk of developing mobile phone dependence by 75%.

Participants with the panic disorder would have significantly more emotional symptoms and dependency on mobile phones than the control group if access to their mobile phones were restricted. In our study, the binary logistic regression results also showed that participants who have reported having anxiety problems or using a treatment for anxiety were at a higher risk of developing mobile phone dependence by 75% [40]. This study discovered intriguing associations between a higher risk of problematic smartphone use and married and divorced respondents than never-married respondents. Being married is associated with a more frequent use of telephone calls, instant messaging, and e-mail for family communication when compared to those who are single, which may contribute to a more reliant use of smartphones [40]. On the contrary, problematic use of smartphone functions unrelated to family communication, such as online video gaming, may exacerbate family relationships and contribute to marital breakdown [40].

The difference between Saudi Arabia's and Jordan's results could be attributed to different samples, economic disparities, personality types, and the impact of other variables such as age and environmental factors. There are also significant economic differences between the nations, influencing smartphone use and the prevalence of smartphone ownership between centuries. As a result, the form of smartphone addiction [41]. Furthermore, it could be because the amount of 4G Wi-Fi coverage in Saudi Arabia has increased [41].

The study is volubility to mental health care results from the correlation between nomophobia and anxiety and stress. Our study aimed to determine the prevalence of nomophobia and its associated factors and recommend initiating a therapeutic strategy and preventing it through awareness programs. Further research should be conducted to ascertain the reasons for this population's increased cell phone use, explore the relationship between nomophobia and other risk factors among university students throughout the kingdom, and support individuals diagnosed with severe nomophobia. We should consider this a critical issue in mental health care, given the numerous connections to psychological problems and the varying consequences for the individual's life. Therefore, with the increasing incidence of stress every year in growing and developed countries, it is vital to focus on the awareness of the general population about the consequences long term dependency on mobile phones, which is one of the critical factors associated with developing anxiety in the modern life.

To our knowledge, no research has been done in Saudi Arabia and Jordan to include all age groups; also, our study has a large sample size among the general population, whereas other studies in the region focus on undergraduate students; and it sheds light on the prevalence of nomophobia, which can aid researchers in assessing nomophobic tendencies and recognizing possible risk factors.

Our study has an unavoidable limitation; firstly, our study is a cross-sectional observational study conducted using a web-based online survey (Twitter, Snapchat, and WhatsApp), and therefore, it might have a recall bias. However, owing to the current epidemic and the nature of the objective of this study, we assume that we targeted a well-representative sample. Secondly, due to the nature of the study design, we could not confirm any association between the predictors and nomophobia. Thirdly, because most participants were between 18 and 29, generalizing the study to all age groups is difficult. Additionally, since that the study was a self-reported online survey and our objectives were about smartphones, it might have induced social desirability bias. In addition, a self-administered questionnaire through an online platform could be biased.

## Conclusion

Our study concludes that the prevalence of nomophobia among the general population in two middle eastern countries is 51.2%. Moreover, age, gender, marital status, and previous history of anxiety may play an essential role in developing mobile phone dependence.

## Data Availability

The datasets used and/or analysed during the current study are available from the corresponding author on reasonable request.

## Abbreviations

SPSS: Statistical Package for Social Sciences
SD: Standard Deviation
ICD: International Classification of Diseases
WHO: World Health Organization
USA: United States of America
COVID: Corona virus diseases
